# Sex, Race, and Age Disparities in the Improvement of Survival for Gastrointestinal Cancer over Time

**DOI:** 10.1038/srep29655

**Published:** 2016-07-13

**Authors:** Jue-feng Wan, Li-feng Yang, Yun-zhu Shen, Hui-xun Jia, Ji Zhu, Gui-chao Li, Zhen Zhang

**Affiliations:** 1 Department of Radiation Oncology, Fudan University Shanghai Cancer, Center, China; 2 Department of Oncology, Shanghai Medical College, Fudan University, Shanghai, China; 3 School of Medicine, Nanjing University, Nanjing, jiangsu, 210093, China; 4 Department of Oncology, Nanjing First Hospital, Nanjing Medical University, Nanjing, jiangsu, 210006, China; 5 Department of Clinical Statistical Center, Fudan University Shanghai Cancer, Center, China

## Abstract

There have been notable improvements in survival over the past 2 decades for gastrointestinal (GI) cancer. However, the degree of improvement by age, race, and sex remains unclear. We analyzed data from 9 population-based cancer registries included in the SEER program of the National Cancer Institute (SEER 9) in 1990 to 2009 (n = 288,337). The degree of survival improvement over time by age, race, and sex was longitudinally measured. From 1990 to 2009, improvements in survival were greater for younger age groups. For patients aged 20 to 49 years and diagnosed from 2005 to 2009, adjusted HRs (95% CIs) were 0.74 (95% CI, 0.66–0.83), 0.49 (95% CI, 0.37–0.64), 0.69 (95% CI, 0.65–0.76), 0.62 (95% CI, 0.54–0.69), and 0.56 (95% CI, 0.42–0.76), for cancer of the stomach, small intestine, colon, rectum and anus, respectively, compared with the same age groups of patients diagnosed during 1990 to 1994. Compared with African Americans, whites experienced greater improvement in small intestinal and anal cancer survival. Female anal cancer and regional anal cancer patients experienced no improvement. Our data suggest that different improvement in survival in age, sex and race exists.

Cancer is a major public health problem in the United States and many other countries. It is currently the second leading cause of death in the United States and gastrointestinal cancer (GI) is one of the most common cancers^1^. There have been notable improvements in survival over the past 2 decades for gastrointestinal cancer attributed to improvements in cancer screening, along with advances in cancer treatments including surgery, radiotherapy, chemotherapy, and targeted therapies survival^2^.

Several cross-sectional studies have compared cancer survival rates by race, sex, and age^3^,^4^,^5^,^6^,^7^,^8^. However, these studies did not address the secular trend of cancer survival, which measures the improvement of cancer survival over time. In this study, we analyzed the differences in the improvement of cancer survival by race, age, and sex in the past 2 decades. We analyzed data from 9 population-based cancer registries included in the SEER program of the National Cancer Institute (SEER 9) in 1990 to 2009 to determine whether improvements in cancer survival differ by race, sex, and age in the United States.

## Methods

### Patient selection in the SEER database

The SEER, a population-based reporting system, was surveyed for the retrospective collection of data used in the analysis. We analyzed data from 9 population-based cancer registries included in the SEER program of the National Cancer Institute (SEER 9). Because no personal identifying information was used in the analysis, this study was granted an exemption from the Institutional Review Board of the study institution on March 30, 2012.

Cases of gastric cancer (C16.0 to C16.9), small intestine cancer (C17.0 to C17.9), colon cancer (C18.0 to C18.9), rectal cancer (C19.9, C20.9) and anal cancer (C21.0, C21.1) from 1990 to 2009 were extracted from the SEER database (SEER*Stat 8.2.1) according to the Site Recode classifications. We excluded patients whose survival months were unknown and SEER historic stage were unknown. Demographic variables (age at diagnosis, year of diagnosis, race, sex, and marital status) and tumor characteristics (stage and histologic types) were obtained from the registry databases.

### Statistical Analysis

The primary outcome in this study was a measure of cancer specific death, defined as a death with the specific cancer. Deaths from other causes were treated as censored observations. Survival rates (at 1, 3, and 5 years) by cancer-specific death by age, sex, or race for patients diagnosed between 1990 and 1994 (the baseline period) were calculated using the Kaplan-Meier method. Hazard ratios (HRs) and 95% CIs for cancer-specific death associated with age, sex, or race were calculated using Cox proportional hazards models, for patients diagnosed during the time periods 1995 to 1999, 2000 to 2004, and 2005 to 2009, and were compared with those diagnosed at the baseline. Cox proportional hazards models were also used to calculate trend tests and derive calculate p-values for improvements in cancer survival. In the final analyses, all models were adjusted for marital status, common histologic types, SEER historic stages (localized, regional, and distant), age (20–49, 50–64, 65–74, or 75–84 years), race (white, African American, or other), and sex. The statistical test was two sided and P < 0.05 was considered statistically significant. PASW Statistics 13 (SPSS Inc., Chicago, USA) was used for the statistical analysis.

## Results

Analyses included 39,997 gastric cancer patients, 10,652 small intestine cancer patients, 165,577 colon cancer patients, 66,945 rectal cancer patients and 5,166 anal cancer patients from 1990 to 2009. During this period, the percentage of cancer cases diagnosed at a localized stage increased for all gastrointestinal cancer sites (Table 1–5 in the Supplement).

The distribution of other patient characteristics also changed over the 20-year study period (Table 1–5 in the Supplement). In general, the percentage of white patients decreased and the percentage of African American patients increased for all gastrointestinal cancer sites except anal cancer. The percentage of 20–49 years patients increased for gastric cancer and colorectal cancer.

For patients diagnosed from 1990 to 1994, survival rates for African Americans were the lowest for colorectal and anal cancer (Table 1). For all cancer sites, survival rates were lowest in the oldest age group (75–84 years) and were lower among men than among women (Table 1).

**Figure 1:**
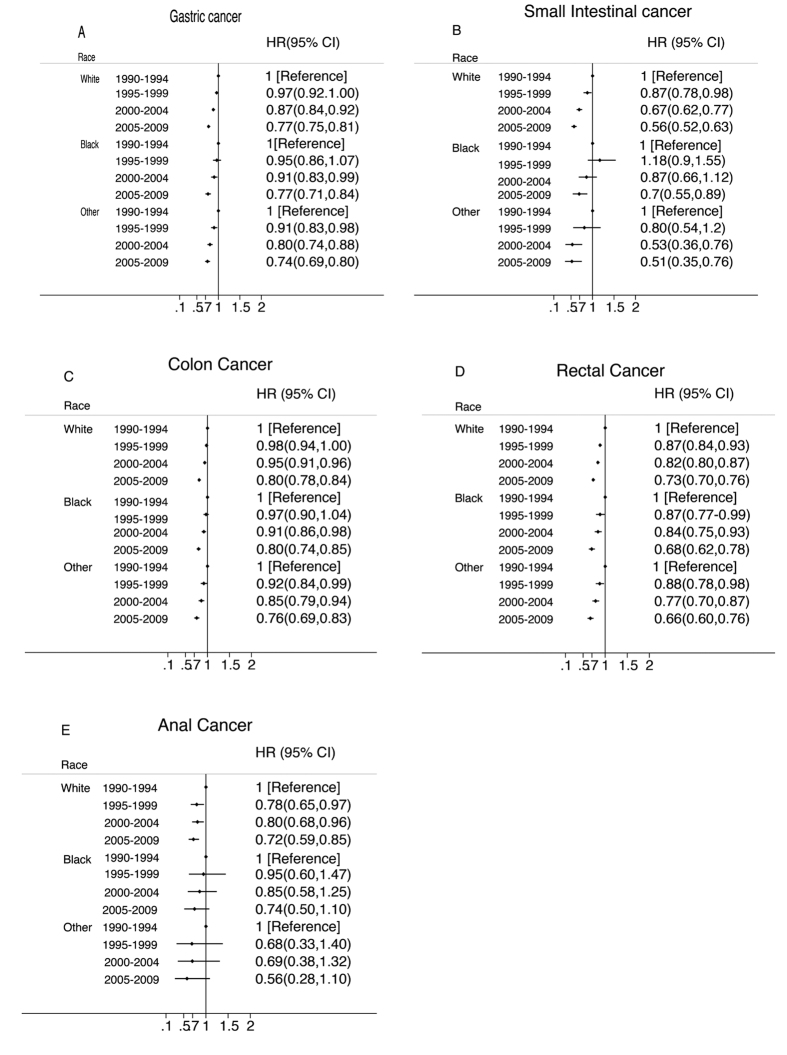
Multivariate-Adjusted Hazard Ratios (HRs) and 95% CIs for Cancer-Specific Death Associated With Year of Diagnosis According to Race, in 9 SEER Registries, 1990–2009. The HRs and 95% CIs were adjusted for marital status, common histologic types, SEER historic stage, age and sex using the time period 1990–1994 as the reference.

Significant improvements in survival, shown by decreasing HRs over time, were observed for all cancers from 1990 to 2009 in all racial groups except for anal cancer (Fig. 1). White and African American cancer patients experienced different degrees of improvement in survival from 1990 to 2009 for small intestinal and anal cancers.

No apparent racial differences in survival improvements were seen for the other cancers evaluated in this study. During the 20-year study period, there was a statistically significant improvement in small intestinal cancer survival among whites and a slight improvement in survival among African Americans. Over the study period, whites experienced a greater improvement in survival of anal cancer than African Americans and African Americans experienced no improvement. There were only 44, 63, 191and 267 African American anal cases in period of 1990–1994, 1995–1999, 2000–2004 and 2005–2009. This small numbers of cases may make it difficult to obtain statistical significance.

**Figure 2:**
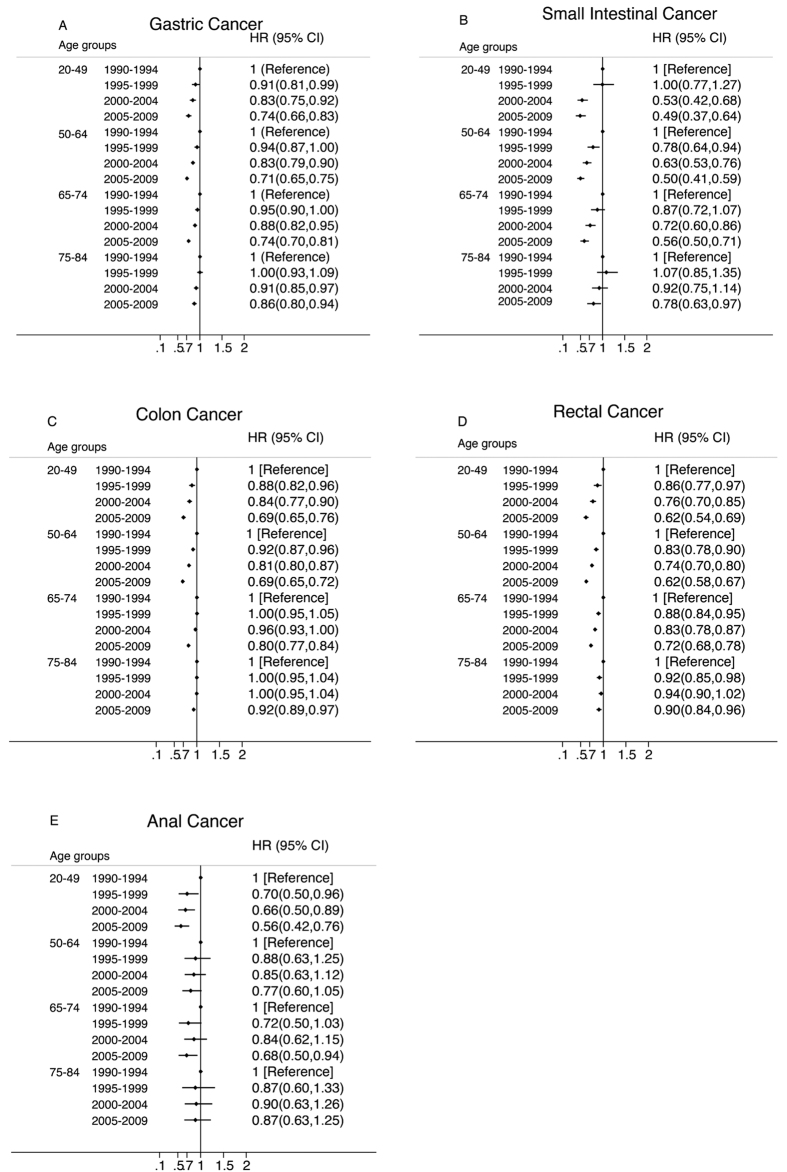
Multivariate-Adjusted Hazard Ratios (HRs) and 95% CIs for Cancer-Specific Death Associated With Year of Diagnosis According to Age, in 9 SEER Registries, 1990–2009. The HRs and 95% CIs were adjusted for marital status, common histologic types, SEER historic stage, race and sex using the time period 1990 to 1994 as the reference.

From 1990 to 2009, all age groups demonstrated improved survival for all cancer sites except for anal cancer (Fig. 2). Improvements in survival were greater for younger age groups. For patients aged 20 to 49 years and diagnosed from 2005 to 2009, adjusted HRs (95% CIs) were 0.74 (95% CI, 0.66–0.83), 0.49 (95% CI, 0.37–0.64), 0.69 (95% CI, 0.65–0.76), 0.62 (95% CI, 0.54–0.69), and 0.56 (95% CI, 0.42–0.76), for cancer of the stomach, small intestine, colon, rectum and anus, respectively, compared with the same age groups of patients diagnosed during 1990 to 1994. However, the corresponding HRs (95% CIs) for elderly patients (those 75–84 years old) were only 0.86 (95% CI, 0.80–0.94), 0.78 (95% CI, 0.63–0.97), 0.92 (95% CI, 0.89–0.97), 0.90 (95% CI, 0.84–0.96), and 0.87 (95% CI, 0.63–1.25), for the same 5 cancer sites, respectively. There were no improvements in anal cancer patients ages 50–64 years and 75–84 years (p = 0.08 and p = 0.56).

**Figure 3:**
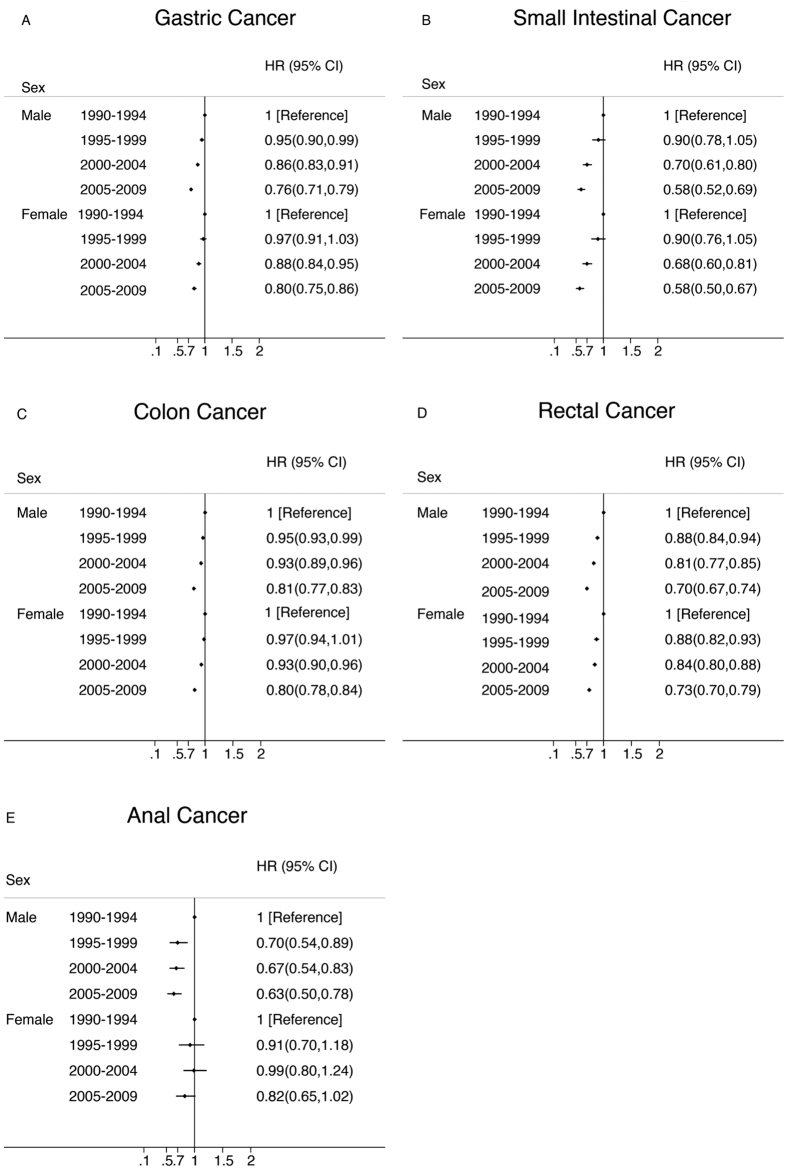
Multivariate-Adjusted Hazard Ratios (HRs) and 95% CIs for Cancer-Specific Death Associated With Year of Diagnosis According to Sex, in 9 SEER Registries, 1990–2009. The HRs and 95% CIs were adjusted for marital status, common histologic types, SEER historic stage, race and age using the time period 1990 to 1994 as the reference.

**Figure 4:**
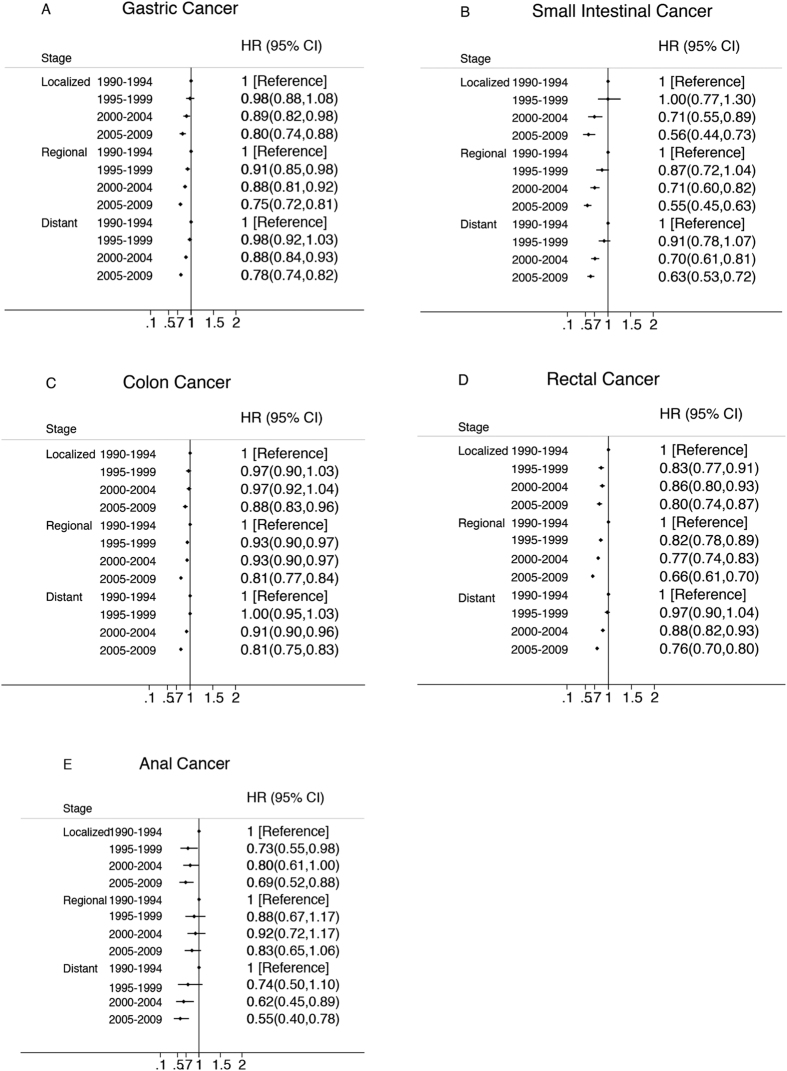
Multivariate-Adjusted Hazard Ratios (HRs) and 95% CIs for Cancer-Specific Death Associated With Year of Diagnosis According to SEER historic stage, in 9 SEER Registries, 1990–2009. HRs and 95% CIs were adjusted for marital status, common histologic types, race, age and sex using the time period 1990–1994 as the reference.

There was a statistically significant improvement in cancer specific survival for both sex and all stages for all cancers studied except for anal cancer (Figs 3 and 4). Female anal cancer and regional anal cancer patients experienced no improvement in this period (p = 0.08 and p = 0.132, respectively). Regional and distant cancers experienced greater improvements than localized cancers in gastric and colorectal cancer.

## Discussion

Using data from the SEER, we have showed a greater improvement in cancer survival over the past 20 years in the United States among younger cancer patients than elderly patients. The widening gap in cancer survival between younger and older patients may be due to differential utilization of newer treatments for elderly patients.

Higher rates of surgical complications, more prevalent co-morbidities, and poorer performance status limit the standard use of multidisciplinary treatment in older patients, and treatment deviation is higher in elderly patients than younger patients. A Surveillance, Epidemiology, and End Results (SEER)–Medicare study of patients more than 65 years of age who were receiving postoperative therapy for rectal cancer showed that 96.6% of Stage III rectal cancer patients completed radiation therapy, only 68.2% and 67.5% completed chemotherapy and both modalities. Among Stage II cancer patients, 91.5% completed radiation therapy but only 49.8% and 47.6% completed chemotherapy and both modalities^9^. Caitlin *et al.* also showed that stage III colon cancer patients who were older received chemotherapy less often and increasing age was associated with lower chemoradiation use among stage II/III rectal cancer patients^10^. In addition, elderly patients were excluded in most clinical trials. Murthy *et al.* showed that there was a strong relationship between age and enrollment fraction, with trial participants 30 to 64 years of age representing 3.0% of incident cancer patients in that age group, in comparison to 1.3% of 65- to 74-year-old patients and 0.5% of patients 75 years of age and older^11^. Lewis *et al.* also found that 32% of participants in clinical trials were elderly, compared with 61% of patients with incident cancers in the United States who were elderly^12^.

Thus, there is insufficient evidence to determine how they will respond to novel targeted therapies or combinations of chemotherapeutic agents. Our findings demonstrate that age-associated disparities exist and this is particularly pressing because this population constitutes the fastest growing subpopulation of cancer patients in the United States.

It has been reported that women may have lower mortality for almost all non–sex-specific cancers than do men^13^,^14^,^15^,^16^. Our study showed that improvements in cancer survival over time were similar between men and women except for anal cancer, for which women had no improvement. We observed a widening gap in survival by race only in small intestine and anal cancer. African Americans experienced no improvement in anal cancer during the 20-year study period. Murphy *et al.* showed that black patients were less likely to receive adjuvant chemotherapy as compared with white patients (risk ratio [RR], .82; 95% CI, .72 to .93)^17^. White *et al.* also suggested that blacks and whites had worse survival that Asians in colorectal cancer^18^. The underlying mechanisms driving this sex and race disparities remain to be investigated.

Regional and distant cancers experienced greater improvements than localized cancers in gastric and colorectal cancer. This is consistent with clinical trial data demonstrating improved survival with improved surgical techniques and novel perioperative treatment for patients with locally advanced and advanced gastric and colorectal cancer^19^,^20^,^21^,^22^,^23^,^24^.

There were several limitations to this study. The population oversamples urban and foreign-born populations, which may affect the generalizability of our findings to the general US population. Other limitations include those inherent in retrospective database analyses and differences in survival expectations between people of different races. Data on individual socioeconomic status, lifestyle factors, insurance and comorbidities were not available, and thus these variables cannot be adjusted in our study. Finally, the small sample size for anal cancer cases was as an explicit limitation of the study.

In conclusion, our data suggest that different improvement in survival in age, sex and race exists. One of the reasons may be differences in GI cancer care across these subpopulations. Finding factors associated with different improvement in GI cancer survival is a key part of improving cancer care for all.

## Additional Information

**How to cite this article**: Wan, J.-f. *et al.* Sex, Race, and Age Disparities in the Improvement of Survival for Gastrointestinal Cancer over Time. *Sci. Rep.* **6**, 29655; doi: 10.1038/srep29655 (2016).

## Supplementary Material

Supplementary Information

## Figures and Tables

**Table 1:** Cancer-Specific Survival Rates for Patients Diagnosed from 1990 to 1994 According to Age Group, Race/Ethnicity, and Sex, in 9 SEER Registries.

Length of Survival, year	Sex		Race			Age			
	Male	Female	White	African American	Other	20–49	50–64	65–74	75–84
Stomach									
1	47.4	51.2	46.2	46.1	55.4	52.3	48.2	48.4	42.9
3	27.2	33.3	26.8	28.2	37.1	28.4	28	31.2	26.5
5	22	28.4	20.9	22.5	31.4	24.3	23	24.5	22.5
Small Intestine									
1	75	77.7	76.3	75	77.2	84.3	76.2	72	66.5
3	58.5	61.2	59.2	57.3	59.1	68.3	61.4	55.9	53.6
5	52	52.5	52.5	53.1	52.4	62.4	55	47.8	43.6
Colon									
1	82	82.3	83.8	78.3	86.4	84.4	84	83.3	78.7
3	69	68.5	68.3	62	72.4	67.4	62.8	71.1	65.6
5	61.4	63.3	62.3	53.7	67.3	61.3	64.2	65.1	60.4
Rectum									
1	86.3	86.6	86	83.2	87.2	88.1	88.4	87	82.2
3	69.7	72	72.7	61.5	74.3	73.4	73	70.5	64.1
5	61.6	64	62	53.3	65.6	66.2	64.2	63.6	55.6
Anal									
1	83.5	91.2	88.6	81.8	84.7	85	93.2	88.8	77.9
3	68.1	79.9	75	66	72.3	69.7	76.1	78.4	67.7
5	60.2	75.2	68.2	58	64.5	62.8	73.3	72.2	58.9
