# Supplementary material for: Sex, Race, and Age Disparities in the Improvement of Survival for Gastrointestinal Cancer over Time
Source: Sci Rep. 2016 Jul 13;6:29655. doi: 10.1038/srep29655 (PMC4942771; doi:10.1038/srep29655)
Supplement: Supplementary Information [file srep29655-s1.pdf]

Sex, Race, and Age Disparities in the Improvement of Survival for Gastrointestinal Cancer over Time

Jue-feng Wan[1,2*], Li-feng Yang[1,2*], Yun-zhu Shen[3,4*], Hui-xun Jia[2,5], Ji Zhu[1,2], Gui-chao Li[1,2], Zhen Zhang[1,2]

[1]Department of Radiation Oncology, Fudan University Shanghai Cancer Center

[2]Department of Oncology, Shanghai Medical College, Fudan University, Shanghai, China

[3]School of Medicine, Nanjing University, Nanjing, jiangsu, China, 210093

[4] Department of Oncology, Nanjing First Hospital, Nanjing Medical University, Nanjing, jiangsu, China, 210006

[5]Department of Clinical Statistical Center, Fudan University Shanghai Cancer Center

*These authors contributed equally to this work.

Corresponding author: Zhen Zhang MD, Department of Radiation Oncology, Fudan University Shanghai Cancer Center, 270 Dong An Road, Shanghai 200032, China.

Email: zhenzhang6@hotmail.com

Work telephone number and work fax number:021-64175590

Table 1. Patient characteristics for gastric cancer from 1990 to 2009 years

Variable	1990-1994	1995-1999	2000-2004	2005-2009	P
Sex					0.035
Male	6292(63.6%)	6363(64.3%)	6334(63.3%)	6650(65.2%)	
Female	3602(36.4%)	3534(35.7%)	3672(36.7%)	3550(34.8%)	
Race					＜0.001
White	6945(70.2%)	6769(68.4%)	7034(70.3%)	6906(67.7%)	
Black	1296(13.1%)	1296(13.1%)	1250(12.5%)	1458(14.3%)	
Other	1653(16.7%)	1831(18.5%)	1722(17.2%)	1836(18.0%)	
Age					＜0.001
20-49	1118(11.3%)	1237(12.5%)	1383(13.8%)	1550(15.2%)	
50-64	2691(27.2%)	2652(26.8%)	2791(27.9%)	3202(31.4%)	
65-74	3235(32.7%)	3266(33.0%)	2821(28.2%)	2887(28.3%)	
75-84	2850(28.8%)	2742(27.7%)	3011(30.1%)	2561(25.1%)	
Stage					＜0.001
Localized	2206(22.3%)	2435(24.6%)	2742(27.4%)	3182(31.2%)	
Regional	3690(37.3%)	3404(34.4%)	3252(32.5%)	3274(32.1%)	
Distant	3998(40.4%)	4058(41.0%)	4012(40.1%)	3744(36.7%)	

Table 2. Patient characteristics for small intestinal cancer from 1990 to 2009 years

Variable	1990-1994	1995-1999	2000-2004	2005-2009	P
Sex					0.233
Male	1077(53.7%)	1353(55.4%)	1513(53.4%)	1760(52.2%)	
Female	930(46.3%)	1089(44.6%)	1320(46.6%)	1610(47.8%)	
Race					0.113
White	1640(81.7%)	1907(78.1%)	2218(78.3%)	2608(77.4%)	
Black	265(13.2%)	349(14.3%)	445(15.7%)	556(6.5%)	
Other	102(5.1%)	186(7.6%)	170(6.0%)	206(6.1%)	
Age					＜0.001
20-49	427(21.3%)	452(18.5%)	505(17.8%)	647(19.2%)	
50-64	612(30.5%)	786(32.2%)	1028(36.3%)	1317(39.1%)	
65-74	580(28.9%)	659(27.0%)	708(25.0%)	680(20.2%)	
75-84	388(19.3%)	545(22.3%)	592(20.9%)	726(21.5%)	
Stage					0.001
Localized	576(28.7%)	769(31.5%)	974(34.4%)	1190(35.3%)	
Regional	773(38.5%)	913(37.4%)	994(35.1%)	1162(34.5%)	
Distant	658(32.8%)	760(31.1%)	865(30.5%)	1018(30.2%)	

Table 3. Patient characteristics for colon cancer from 1990 to 2009 years

Variable	1990-1994	1995-1999	2000-2004	2005-2009	P
Sex					0.038
Male	20601(50.4%)	21418(51.3%)	21517(50.5%)	21017(52.1%)	
Female	20275(49.6%)	20333(48.7%)	21092(49.5%)	19324(47.9%)	
Race					＜0.001
White	34132(83.5%)	33693(80.7%)	34257(80.4%)	31264(77.5%)	
Black	4087(10.0%)	4509(10.8%)	5241(12.3%)	5123(12.7%)	
Other	2657(6.5%)	3549(8.5%)	3111(7.3%)	3954(9.8%)	
Age					＜0.001
20-49	3352(8.2%)	3966(9.5%)	4815(11.3%)	4881(12.1%)	
50-64	10137(24.8%)	10646(25.5%)	11973(28.1%)	13514(33.5%)	
65-74	14593(35.7%)	14029(33.6%)	12527(29.4%)	10287(25.5%)	
75-84	12794(31.3%)	13110(31.4%)	13294(31.2%)	11659(28.9%)	
Stage					＜0.001
Localized	14388(35.2%)	15656(37.5%)	16276(38.2%)	17225(42.7%)	
Regional	16146(39.5%)	17243(41.3%)	16362(38.4%)	14160(35.1%)	
Distant	10342(25.3%)	8852(21.2%)	9971(23.4%)	8956(22.2%)	

Table 4. Patient characteristics for rectal cancer from 1990 to 2009 years

Variable	1990-1994	1995-1999	2000-2004	2005-2009	P
Sex					0.337
Male	9103(57%)	9786(58%)	9755(57.4%)	9990(58.4%)	
Female	6868(43%)	7087(42%)	7240(42.6%)	7116(41.6%)	
Race					＜0.001
White	13288(83.2%)	13718(81.3%)	13834(81.4%)	13308(77.8%)	
Black	1262(7.9%)	1366(8.1%)	1666(9.8%)	1967(11.5%)	
Other	1421(8.9%)	1789(10.6%)	1495(8.8%)	1831(10.7%)	
Age					＜0.001
20-49	1869(11.7%)	2396(14.2%)	2634(15.5%)	3130(18.3%)	
50-64	4855(30.4%)	5433(32.2%)	5880(34.6%)	7031(41.1%)	
65-74	5366(33.6%)	5078(30.1%)	4860(28.6%)	4054(23.7%)	
75-84	3881(24.3%)	3966(23.5%)	3621(21.3%)	2891(16.9%)	
Stage					＜0.001
Localized	6899(43.2%)	7643(45.3%)	8140(47.9%)	8314(48.6%)	
Regional	5829(36.5%)	6260(37.1%)	6016(35.4%)	5987(35.0%)	
Distant	3243(20.3%)	2970(17.6%)	2839(16.7%)	2805(16.4%)	

Table 5. Patient characteristics for anal cancer from 1990 to 2009 years

Variable	1990-1994	1995-1999	2000-2004	2005-2009	P
Sex					0.002
Male	357(43.5%)	500(46.3%)	578(42.2%)	796(42%)	
Female	464(56.5%)	580(53.7%)	791(57.8%)	1100(58%)	
Race					0.055
White	734(89.4%)	950(88%)	1142(83.4%)	1551(81.8%)	
Black	44(5.4%)	63(5.8%)	191(14.0%)	267(14.1%)	
Other	43(5.2%)	67(6.2%)	36(2.6%)	78(4.1%)	
Age					＜0.001
20-49	194(23.6%)	340(31.5%)	432(31.6%)	483(25.5%)	
50-64	258(31.4%)	353(32.7%)	517(37.8%)	861(45.4%)	
65-74	221(27%)	243(22.5%)	263(19.2%)	315(16.6%)	
75-84	148(18%)	144(13.3%)	157(11.4%)	237(12.5%)	
Stage					＜0.001
Localized	438(53.3%)	602(55.8%)	773(56.5%)	1115(58.8%)	
Regional	283(34.5%)	360(33.3%)	436(31.8%)	620(32.7%)	
Distant	100(12.2%)	118(10.9%)	160(11.7%)	161(8.5%)	
